# Principal component analysis and the locus of the Fréchet mean in the space of phylogenetic trees

**DOI:** 10.1093/biomet/asx047

**Published:** 2017-09-27

**Authors:** Tom M W Nye, Xiaoxian Tang, Grady Weyenberg, Ruriko Yoshida

**Affiliations:** 1 *School of Mathematics and Statistics, Newcastle University, Newcastle upon Tyne NE1 7RU, U.K.* tom.nye@ncl.ac.uk; 2 *Department of Mathematics, Texas A&M University, College Station, Texas 77843, U.S.A.* xiaoxian@math.tamu.edu; 3 *Department of Mathematics, University of Hawaii at Hilo, Hilo, Hawaii 96720, U.S.A.* gradysw@hawaii.edu; 4 *Department of Operations Research, Naval Postgraduate School, Monterey, California 93943, U.S.A.* ryoshida@nps.edu

**Keywords:** Fréchet mean, Phylogenetic tree, Principal component analysis, Tree space

## Abstract

Evolutionary relationships are represented by phylogenetic trees, and a phylogenetic analysis of gene sequences typically produces a collection of these trees, one for each gene in the analysis. Analysis of samples of trees is difficult due to the multi-dimensionality of the space of possible trees. In Euclidean spaces, principal component analysis is a popular method of reducing high-dimensional data to a low-dimensional representation that preserves much of the sample’s structure. However, the space of all phylogenetic trees on a fixed set of species does not form a Euclidean vector space, and methods adapted to tree space are needed. Previous work introduced the notion of a principal geodesic in this space, analogous to the first principal component. Here we propose a geometric object for tree space similar to the $k$th principal component in Euclidean space: the locus of the weighted Fréchet mean of $k+1$ vertex trees when the weights vary over the $k$-simplex. We establish some basic properties of these objects, in particular showing that they have dimension $k$, and propose algorithms for projection onto these surfaces and for finding the principal locus associated with a sample of trees. Simulation studies demonstrate that these algorithms perform well, and analyses of two datasets, containing Apicomplexa and African coelacanth genomes respectively, reveal important structure from the second principal components.

## 1. Introduction

A great opportunity offered by modern genomics is that phylogenetics applied on a genomic scale, or phylogenomics, should be especially powerful for elucidating gene and genome evolution, relationships among species and populations, and processes of speciation and molecular evolution. However, a well-recognized hurdle is the sheer volume of genomic data that can now be generated relatively cheaply and quickly, but for which analytical tools are lacking. There is a major need to explore new approaches that will enable us to undertake comparative genomic and phylogenomic studies much more rapidly and robustly than existing tools allow.

Datasets consisting of collections of phylogenetic trees are challenging to analyse, due to their high dimensionality and the complexity of the space containing the data. Multivariate statistical procedures such as outlier detection (Weyenberg et al., 2014), clustering (Gori et al., 2016) and multi-dimensional scaling (Hillis et al., 2005) have previously been applied to such datasets, but principal component analysis is perhaps the most useful multivariate statistical tool for exploring high-dimensional datasets. For example, Zha et al. (2001) and Ding & He (2004) showed that principal component analysis automatically projects to the subspace where the global solution of $K$-means clustering lies, and so facilitates $K$-means clustering to find near-optimal solutions. Although principal component analysis for data in ${\mathbb{R}}^m$ can be defined in several different ways, the following description is natural for reformulating the procedure in tree space. Suppose we have data $Z=\{z_1,\ldots,z_n\}$ where $z_i\in{\mathbb{R}}^m$ for $i=1,\ldots,n$. For any set of $k+1$ points $V=\{v_0,\ldots,v_k\}\subset{\mathbb{R}}^m$ we can define

(1) \begin{equation*}\label{equ:def_euc_PC} \Pi(V)=\left\{ \sum_{i=0}^k p_iv_i: p_0,\ldots,p_k\in{\mathbb{R}},\: p_0+\cdots+p_k=1 \right\}, \end{equation*}

so that $\Pi(V)$ is the affine subspace of ${\mathbb{R}}^m$ containing $v_0,\ldots,v_k$. The orthogonal $L^2$ distance of any point $y\in{\mathbb{R}}^m$ from $\Pi(V)$ is denoted by $d\{y,\Pi(V)\}$, and the sum of squared projected distances of the data $Z$ onto $\Pi(V)$ is denoted by

\begin{equation*}
D^2_{Z}\{\Pi(V)\} = \sum_{i=1}^n d\{z_i,\Pi(V)\}^2\text{.}
\end{equation*}

Then the $k$th principal component $\Pi_k$ corresponds to a choice of $V$ which minimizes this sum. In ${\mathbb{R}}^m$, $\:\Pi_0$ is the sample mean, $\Pi_1$ is the line through the sample mean which minimizes the sum of squared projected distances, and so on for $k=2,3,\ldots$. Although it is not explicit in the definition above, in ${\mathbb{R}}^m$ the principal components are nested, i.e., $\Pi_0\subset\Pi_1\subset\Pi_2\subset\cdots$. This description of principal component analysis relies heavily on the vector space properties of ${\mathbb{R}}^m$: $\Pi(V)$ is defined as a linear combination of vectors and the procedure uses orthogonal projection.

However, the space of phylogenetic trees on a fixed set of leaves is not a Euclidean vector space, so we cannot directly apply classical principal component analysis to a dataset of phylogenetic trees. Instead, Billera et al. (2001) showed that the set $\mathcal{T}_{N}$ of all phylogenetic trees with $N+1$ leaves labelled $0,1,\ldots,N$ forms a CAT$(0)$ space as defined by Bridson & Haefliger (2011, Definition II.1.1). In CAT$(0)$ spaces any pair of points are joined by a unique geodesic, or shortest-length path, and an algorithm exists that computes $\mathcal{T}_{N}$ geodesics in $O(N^4)$ steps (Owen & Provan, 2011). Furthermore, projection onto closed sets is well defined in CAT$(0)$ spaces.

The analogue of the zeroth principal component is the unweighted Fréchet mean of the data $z_1,\ldots, z_n$. The Fréchet mean is a statistic which characterizes the central tendency of a distribution in arbitrary metric spaces. For any metric space $S$ equipped with metric $d(\cdot\,, \cdot)$, the Fréchet population mean $\mu$ with respect to the distribution $\nu$ is defined by

\begin{equation*}
\mu(\nu) = \mathop{\mathrm{arg\,min}}\limits_{y\in S} \int_S d(y,x)^2 \,{\rm d}\nu(x)\text{.}
\end{equation*}

The discrete analogue, the weighted Fréchet mean of a sample $Z=\{z_1,\ldots, z_n\}$ with respect to a weight vector $w$, is

\begin{equation*}
\mu(Z,w) = \mathop{\mathrm{arg min}}\limits_{y\in S} \sum_{i=1}^n w_i\,d(y,z_i)^2,
\end{equation*}

where the weights $w_i$ satisfy $w_i\geq 0$ for $i = 1, \ldots, n$. In any CAT$(0)$ space, $\mu(Z,w)$ is a well-defined unique point given data $Z$ and weight vector $w$. The definition of the zeroth principal component $\Pi_0$ in ${\mathbb{R}}^m$ given above coincides with the definition of the Fréchet sample mean with weights $w_i=1$ in any CAT$(0)$ space. Several algorithms for computing the Fréchet sample mean in $\mathcal{T}_{N}$ have been developed (Bačák, 2014; Miller et al., 2015) and we review these in § 2.2, as they play an important role in our method. The term Fréchet mean will be used throughout to refer to a sample mean unless stated otherwise.

Methods for constructing a principal geodesic in tree space, an analogue of $\Pi_1\subset{\mathbb{R}}^m$ as defined above, have recently been developed. In Nye (2011), the approach involved firing geodesics from some mean tree. For each candidate geodesic $\Gamma$, the sum of squared projected distances $D^2_Z(\Gamma)$ was computed and a greedy algorithm was used to adjust $\Gamma$ in order to minimize $D^2_Z(\Gamma)$. The geodesics considered were infinitely long, but have the disadvantage that in some cases many such geodesics fit the data equally well. Subsequent approaches therefore considered finitely long geodesic segments (Feragen et al., 2013; Nye, 2014). The geodesic segment between two points $v_0,v_1\in\mathcal{T}_{N}$ is analogous to $\Pi(V)$ in (1) with $k=1$, except that the weights $p_0$ and $p_1$ must be constrained to be a valid probability vector; that is, $p_0$ and $p_1$ must be nonnegative and sum to 1. Feragen et al. (2013) constrained the ends of the geodesic to be points in the sample $Z$ and sought the corresponding geodesic $\Gamma$ which minimizes $D^2_Z(\Gamma)$, whereas Nye (2014) did not restrict the geodesic and used a stochastic optimization algorithm to perform the minimization.

In this paper we address two fundamental questions: (i) which geometric object most naturally plays the role of a $k$th principal component in tree space; and (ii) given such an object, how can we efficiently project data points onto the object? Our proposed solution is to replace the definition of $\Pi(V)\subset{\mathbb{R}}^m$ given in (1) with the locus of the weighted Fréchet mean of points $v_0,\ldots,v_k$ in tree space. Specifically, suppose $V=\{v_0,\ldots,v_k: v_i\in\mathcal{T}_{N}, \,i=0,\ldots,k\}$ and define $\Pi(V)\subset\mathcal{T}_{N}$ by

\begin{equation*}
\Pi(V) = \{ \mu(V,p) : p\in\mathcal{S}^{k}\}
\end{equation*}

where $\mathcal{S}^{k}$ is the $k$-dimensional simplex of probability vectors,

\begin{equation*}
\mathcal{S}^{k} = \left\{(p_0,\ldots,p_k): p_i\geq 0, \,i=0,\ldots,k,
\,\sum_{i=0}^k p_i=1\right\}\!,
\end{equation*}

and $\mu(V,p)$ is the Fréchet mean of the points in set $V$ with weights $p$. We call $\Pi(V)$ the locus of the Fréchet mean of $V$. Our choice of notation is intended to emphasize the analogy between the definition of $\Pi(V)$ in tree space and the corresponding definition for ${\mathbb{R}}^m$ in (1). The locus of the Fréchet mean is a type of minimal surface, as the following physical analogy suggests. Imagine connecting a point $y\in\mathcal{T}_{N}$ to points $v_0,\ldots,v_k\in\mathcal{T}_{N}$ by $k+1$ pieces of elastic. When the point $y$ is free to move, it will move under the action of the elastic into an equilibrium position in tree space. If the stiffness of each piece of elastic is allowed to vary independently, corresponding to different choices for $p\in\mathcal{S}^{k}$, the equilibrium point will move about in tree space, tracing out a surface. In Euclidean space the locus of the Fréchet mean of some collection of points is an affine subspace; however, in tree space, the locus can be curved. Surfaces of this kind have recently been studied in the context of Riemannian manifolds and other geodesic metric spaces (Pennec, 2015). We discuss the relationship of the present paper to that work in § 6.

Our main theoretical results are as follows. First, when $V=\{v_0,\ldots,v_k\}$ we derive a set of local implicit equations for $\Pi(V)$. These allow us to derive conditions for $\Pi(V)$ to be locally flat, and also enable us to construct explicit realizations of $\Pi(V)$ in certain cases. Secondly, using the implicit equations we show that the locus of the Fréchet mean $\Pi(V)$ in $\mathcal{T}_{N}$ is locally $k$-dimensional for generic nondegenerate choices of $V$, and thus forms a suitable candidate for a $k$th principal component. Third, we present an algorithm for projection onto $\Pi(V)$ which relies only on the CAT$(0)$ properties of $\mathcal{T}_{N}$. We demonstrate accuracy of the projection algorithm via a simulation study.

## 2. The geometry of tree space

### 2.1. Construction of tree space and its geodesics

Throughout the paper, the $m$-dimensional Euclidean vector space is denoted by ${\mathbb{R}}^m$. The nonnegative and positive orthants in ${{\mathbb{R}}}^m$ are denoted by ${\mathbb{R}}_{\geq 0}^m$ and ${\mathbb{R}}_{>0}^m$, respectively. For any vectors $x,y\in {{\mathbb{R}}}^m$, $\|x\|$ denotes the Euclidean norm of $x$ and $\langle x,y\rangle$ denotes the Euclidean inner product.

A phylogenetic tree with leaf set $X=\{0,1,\ldots,N\}$ is an undirected weighted acyclic graph with $N+1$ degree-$1$ vertices labelled $0,1,\ldots,N$ and with no degree-$2$ vertices. We consider rooted trees, and the root is the leaf labelled 0. Each such tree contains $N+1$ pendant edges, which connect to the leaves, and up to $N-2$ internal edges. The maximum number of internal edges is achieved when the tree is binary, in which case all non-leaf vertices have degree $3$, and the tree is said to be fully resolved. If a tree contains fewer edges, then it is said to be unresolved and there must be at least one vertex with degree $4$ or higher. Apart from the root edge containing taxon $0$, each edge in a phylogeny is assigned a strictly positive weight, also called the edge length. Given a tree $x\in\mathcal{T}_{N}$, the set of edges of $x$ is denoted by ${\mathcal{E}}({x})$, and the weight assigned to $e\in{\mathcal{E}}({x})$ is denoted by $|e|_x$. It is convenient to define $|e|_x$ to be zero whenever $e$ is not contained in $x$.

Tree space $\mathcal{T}_{N}$ is the set of all phylogenetic trees with leaf set $X$ (Billera et al., 2001). Tree space can be embedded in ${\mathbb{R}}^M$ for $M={2^N-1}$ in the following way. If we cut any edge $e\in{\mathcal{E}}({x})$, then the tree $x$ splits into two disconnected pieces. This determines a split $X_e|{X}^{\rm c}_e$ of the leaf set $X$, where $X_e\cup{X}^{\rm c}_e=X$ and $X_e\cap{X}^{\rm c}_e=\emptyset$. By convention we choose $X_e$ to be the set containing the root 0, and so there are $M=2^N-1$ possible splits of $X$. The collection of splits represented by a tree $x$ is called the topology of $x$. Since edges and splits are equivalent, we use the notation ${\mathcal{E}}({x})$ to also represent the set of splits in $x$. By choosing some arbitrary ordering of the set of all splits, each tree $x\in\mathcal{T}_{N}$ can be represented as a vector in ${\mathbb{R}}^M$ with up to $2N-2$ positive entries given by the edge weights of $x$ and zeros for each split that is not contained in $x$. However, an arbitrary choice of vector will not necessarily represent a tree; for example, the splits $\{0,1\}|\{2,3,\ldots,N\}$ and $\{0,2\}|\{1,3,\ldots,N\}$ cannot both be contained in the same tree, so any vector for which these splits both have a strictly positive value does not represent a tree. Two splits $X_e|{X}^{\rm c}_e$ and $ X_f|{X}^{\rm c}_f$ are compatible if one of the four sets $X_e \cap X_f$, ${X}^{\rm c}_e \cap X_f$, $X_e \cap {X}^{\rm c}_f$ and ${X}^{\rm c}_e \cap {X}^{\rm c}_f$ is empty, in which case there is at least one tree containing both splits. Any collection of pairwise compatible splits determines a valid tree topology (Semple & Steel, 2003, Theorem 3.1.4).

The embedding into Euclidean space reveals the combinatorial structure of $\mathcal{T}_{N}$. Every tree $x\in\mathcal{T}_{N}$ contains $N$ pendant edges other than the root edge, so $\mathcal{T}_{N}$ is the product of ${\mathbb{R}}^N_{>0}$ and a space corresponding to the internal edges. It is therefore convenient to ignore the pendant edges and consider the corresponding embedding of tree space into $\mathcal{R}_N = {\mathbb{R}}^{M-N}$. Given any tree topology $\tau$ containing $m$ internal edges, the set of trees with topology $\tau$ corresponds to a subset ${\mathcal{O}}_\tau\subset\mathcal{R}_N$ which is isomorphic to ${\mathbb{R}}^{m}_{>0}$ with respect to the local Euclidean structure. Each such region is called the orthant for topology $\tau$. The boundary of ${\mathcal{O}}_\tau$ in $\mathcal{R}_N$ corresponds to trees obtained by removing one or more internal edges from $\tau$. Equivalently, the trees on the boundary can be obtained by taking a tree $x$ in ${\mathcal{O}}_\tau$ and continuously shrinking one or more internal edges down to length zero. Thus, for a fully resolved topology $\tau$, the codimension-$1$ boundaries of ${\mathcal{O}}_\tau$ correspond to trees containing $N-3$ internal edges, and in general each codimension-$k$ boundary corresponds to trees containing $N-k-2$ internal edges, for $k=1,\ldots,N-2$. There are $(2N-3)!!$ possible fully resolved rooted tree topologies, and so $\mathcal{T}_{N}$ is built from $(2N-3)!!$ orthants isomorphic to ${\mathbb{R}}^{N-2}_{>0}$ together with the boundaries of these orthants which correspond to trees that are not fully resolved. Orthants are glued together at their boundaries, since a given unresolved tree containing $m$ internal edges can be obtained by removing edges from several different trees containing $m+1$ edges. Orthants corresponding to fully resolved topologies are glued at their codimension-$1$ boundaries in a relatively simple way. If a single internal edge in a tree with fully resolved topology $\tau$ is contracted to length zero and removed from the tree, the result is a vertex of degree $4$. There are three possible ways to add in an extra edge to give a fully resolved topology, so each codimension-$1$ face of ${\mathcal{O}}_\tau$ is glued to two other such orthants. Trees containing no internal edges are called star trees; the point $0\in\mathcal{R}_N$ corresponds to the set of star trees and is contained in the boundary of every orthant ${\mathcal{O}}_\tau$.

The topology of $\mathcal{T}_{N}$ is taken to be that induced by the embedding into Euclidean space. Geodesics are constructed by considering continuous paths in $\mathcal{T}_{N}$ which are Euclidean straight-line segments in each orthant. The length of a path is the sum of the Euclidean segment lengths. As shown by Billera et al. (2001), the shortest such path or geodesic between two points $x,y\in\mathcal{T}_{N}$ is unique, and it will be denoted by $\Gamma(x,y)$. The distance $d(x,y)$ is defined to be the length of $\Gamma(x,y)$, and this defines the metric $d(\cdot\,,\cdot)$ on $\mathcal{T}_{N}$. By definition, $d(x,y)$ incorporates information about both the topologies and the edge lengths of $x$ and $y$. Given two points $x$ and $y$ in the same orthant, $\Gamma(x,y)$ is simply the Euclidean line segment between $x$ and $y$, whereas when $x$ and $y$ are in different orthants, $\Gamma(x,y)$ consists of a series of straight-line segments traversing orthants corresponding to different topologies. Billera et al. (2001) proved that $\mathcal{T}_{N}$ is a CAT$(0)$ space, so it has several additional geometrical properties (Bridson & Haefliger, 2011).

Owen & Provan (2011) established an $O(N^4)$ algorithm to compute the geodesic between any two trees in $\mathcal{T}_{N}$. The details of their algorithm are not important for the present application, but we do require some notation for the form of the geodesics it constructs. Given $x,y\in\mathcal{T}_{N}$, let $\mathcal{C}(x,y)$ be the set of splits in ${\mathcal{E}}({x})\cup{\mathcal{E}}({y})$ which are compatible with every split in ${\mathcal{E}}({x})$ and every split in ${\mathcal{E}}({y})$. Adopting notation from Owen & Provan (2011), the geodesic $\Gamma(x,y)$ is characterized by disjoint sets of internal splits

\begin{equation*}
A_{xy}^{(1)}, \ldots, A_{xy}^{(\ell_{xy})} \subset {\mathcal{E}}({x}),\quad B_{xy}^{(1)}, \ldots, B_{xy}^{(\ell_{xy})} \subset {\mathcal{E}}({y}),
\end{equation*}

where $\ell_{xy}\geq0$ is an integer that depends on $x$ and $y$. These sets of splits determine the order in which edges are removed and added as the geodesic is traversed; the $j$th topology visited contains splits

\begin{equation*}
B^{(1)}_{xy} \cup \cdots \cup B^{(j)}_{xy} \cup A^{(j+1)}_{xy} \cup
\cdots\cup A^{(\ell_{xy})}_{xy}\cup \mathcal{C}(x,y)\quad
(j=0,\ldots,\ell_{xy})\text{.}
\end{equation*}

The union $\bigcup A_{xy}^{(j)}$$(j=1,\ldots,\ell_{xy})$ is ${\mathcal{E}}({x})\setminus\mathcal{C}(x,y)$ and similarly for tree $y$. We let $\mathcal{A}(x,y)$ be the ordered list of sets $(A_{xy}^{(j)}:j=1,\ldots,\ell_{xy})$ and similarly define $\mathcal{B}(x,y)$. The support of $\Gamma(x,y)$, defined to be the triple $\{\mathcal{A}(x,y),\mathcal{B}(x,y),\mathcal{C}(x,y)\}$, characterizes the sequence of orthants the geodesic traverses. For any set $E\subset{\mathcal{E}}({x})$ we adopt the notation

\begin{equation*}
\|E\|_x = \left( \sum_{e\in E} |e|_x^2\right)^{1/2},
\end{equation*}

and similarly for subsets of ${\mathcal{E}}({y})$. Owen & Provan (2011) showed that

(2) \begin{equation*}\label{equ:geodlen} d(x,y)^2 = \|A_{xy}+B_{xy}\|^2 + \|C_{xy}-D_{xy}\|^2, \end{equation*}

where $A_{xy}$ is the $\ell_{xy}$-dimensional vector whose $j$th element is $\|A_{xy}^{(j)}\|_x$, and similarly for $B_{xy}$ the $j$th element is $\|B_{xy}^{(j)}\|_y$. The vectors $C_{xy}$ and $D_{xy}$ have dimension $|\mathcal{C}(x,y)|$ and respectively contain the edge lengths $|e|_x$ and $|e|_y$ for $e\in \mathcal{C}(x,y)$. It follows from (2) that

(3) \begin{equation*}\label{equ:geod_dist} d(x,y)^2 = \|x\|^2+\|y\|^2+2\langle A_{xy}, B_{xy} \rangle-2\langle C_{xy}, D_{xy} \rangle, \end{equation*}

where $\|x\|^2$ is the sum of squared edge lengths in $x$ and similarly for $y$.

The following definition characterizes certain geodesics which behave rather like Euclidean straight lines.

Definition 1 (Simple geodesic). *Suppose that $x,y\in\mathcal{T}_{N}$ are fully resolved. The geodesic $\Gamma(x,y)$ is said to be simple if each of the sets $A_{xy}^{(i)}$ and $B_{xy}^{(i)}$ contains exactly one element for $i=1,\ldots,\ell_{xy}$. Equivalently, $\Gamma(x,y)$ is simple if and only if at most one edge length at a time contracts to zero as the geodesic is traversed.*

The following definition determines the set of trees $y$ such that the geodesics $\Gamma(x,y)$ to a fixed point $x$ all share the same support.

Definition 2 (Support region). *Fix some point $x\in\mathcal{T}_{N}$ and an orthant ${\mathcal{O}}_\tau$ corresponding to a fully resolved topology $\tau$. Let $\sigma$ be the support of $\Gamma(x,z)$ for some $z\in{\mathcal{O}}_\tau$. Then the set*

\begin{equation*}
S_x(\sigma,\tau)=\{y\in{\mathcal{O}}_\tau:\Gamma(x,y) {\it{has\,\,support}} \sigma\}
\end{equation*}

*is called a support region. The number of support regions for fixed $x$ and $\tau$ is finite since geodesics of the form $\Gamma(x,z)$ for $z\in{\mathcal{O}}_\tau$ have finitely many distinct supports.*

Miller et al. (2015) considered very similar subsets of $\mathcal{T}_{N}$ and established their properties. This relied on a map $\mathcal{T}_{N}\rightarrow\mathcal{T}_{N}$ defined by squaring edge lengths. In the image of this map, Miller et al. (2015) showed that each support region is defined by a set of linear inequalities and that the boundaries between support regions are codimension-$1$ hyperplanes. It follows, by inverting the squaring map, that the union over the set $\Sigma_{x,\tau}$ of possible supports, $\bigcup_{\sigma\in\Sigma_{x,\tau}} S_x^\circ(\sigma,\tau)$, is dense in ${\mathcal{O}}_\tau$, where $S_x^\circ(\sigma,\tau)$ denotes the interior of each support region; it also follows that the boundaries between the support regions are continuous codimension-$1$ surfaces within each orthant.

### 2.2. Algorithms for computing the Frechét mean

Several algorithms for computing the unweighted or weighted Fréchet mean of a sample in $\mathcal{T}_{N}$ have been developed (Sturm, 2003; Bačák, 2014; Miller et al., 2015). These algorithms have the following general structure. Suppose we have a set $V=\{v_0,\ldots,v_k\}\subset\mathcal{T}_{N}$. At the $i$th iteration there is an estimate $\mu_i$ of the Fréchet mean of $V$. To find the next estimate, $\mu_{i+1}$, a data point $v_j$ is selected, either deterministically or stochastically depending on the particular algorithm. The geodesic $\Gamma(\mu_i, v_j)$ is constructed, and $\mu_{i+1}$ is taken to be the point a certain proportion of the distance along the geodesic. This proportion can depend on the weights when the weighted Fréchet mean is estimated. In each case, some form of convergence of the sequence $\mu_0,\mu_1,\mu_2,\ldots$ to the Fréchet mean of $V$ can be proved, independent of the initial estimate $\mu_0$.

Our method does not make direct use of these algorithms. However, as described in § 4.1, our proposed algorithm for projecting data onto the locus of the Fréchet mean is adapted from the algorithm of Sturm (2003), which computes the Fréchet mean of $v_0,\ldots,v_k$ using weights $p_0,\ldots,p_k\geq0$. By definition, the Fréchet mean is invariant under positive scaling of the weights, so we can assume $p_0+\cdots+p_k=1$ without loss of generality. Sturm’s algorithm proceeds in the following way.

Algorithm 1. Sturm’s algorithm for the weighted Fréchet mean.

Fix an initial estimate $\mu_0$ and set $i=0$.

Repeat:

  Sample $V_i\in\{v_0,\ldots,v_k\}$ such that ${\mathrm{pr}}\left({V_i = v_j}\right) = p_j$.

  Construct $\Gamma(\mu_i, V_i)$.

  Let $\mu_{i+1}$ be the point a proportion $s_i$ along $\Gamma(\mu_i, V_i)$, where $s_i=1/(i+2)$.

  Set $i\leftarrow i+1$.

Until the sequence $\mu_0,\mu_1,\ldots$ converges.

Convergence can be tested in various ways, for example by repeating until a specified number of consecutive estimates $\mu_i$ all lie within distance $\epsilon$ of each other. Sturm proved that the points $\mu_i$ converge in probability to the Fréchet mean of the distribution defined by sampling $v_0,\ldots,v_k$ according to probabilities $p_0,\ldots,p_k$.

The deterministic algorithm of Bačák (2014) for computing the weighted Fréchet mean is similar to Sturm’s algorithm, except that the data points are used cyclically, as opposed to being randomly sampled, and the weighting is instead taken into account in the definition of the proportions $s_i$. We use the algorithm of Bačák (2014) for computing the Fréchet mean in order to test our projection algorithm, and this procedure is also described in § 4.1.

### 2.3. Convex hulls

Nye (2014) suggested that the convex hull of $k+1$ points in $\mathcal{T}_{N}$ might be a suitable geometrical object to represent a $k$th principal component. A set $A\subset\mathcal{T}_{N}$ is convex if and only if for all points $x,y\in A$ the geodesic $\Gamma(x,y)$ is also contained in $A$. The convex hull of a set of points is the smallest convex set containing those points. Any geodesic segment is the convex hull of its endpoints, and using the convex hull of three points to represent a second principal component is a natural generalization of the idea of a principal geodesic. Convexity is also a desirable property when performing projections, as occurs in principal component analysis. However, convex hulls in tree space do not have the correct dimension. Examples for which the convex hull of three points is three-dimensional can readily be constructed, as shown in a 2015 University of Kentucky PhD thesis by G. Weyenberg and in Lubiw et al. (2017). Lin et al. (2016, § 3) show that the dimension of a convex hull of three points in $\mathcal{T}_{N}$ can be arbitrarily high as $N$ increases. More generally, convex hulls in tree space are difficult to characterize geometrically, and several fundamental questions remain unanswered. These issues make convex hulls less appealing as geometrical objects to represent principal components, so we focus our attention on the locus of the Fréchet mean. We shall, however, demonstrate the relationship between the locus of the Fréchet mean and the convex hull for an explicit configuration of three points $v_0,v_1,v_2\in\mathcal{T}_{N}$ later in § 3.4.

## 3. The locus of the Fréchet mean

### 3.1. Basic properties

Throughout this section we work with $k+1$ vertex points $v_0,\ldots,v_k\in\mathcal{T}_{N}$ and let $V=\{v_0,\ldots,v_k\}$. As in § 1, we define $\mu:(\mathcal{T}_{N})^{k+1}\times\mathcal{S}^{k}\rightarrow\mathcal{T}_{N}$ by

\begin{equation*}
\mu(V,p) =\mathop {\mathrm{arg min}}\limits_{x\in \mathcal{T}_{N}} \sum_{i=0}^k p_i\,d(x,v_i)^2
\end{equation*}

and denote the associated locus of the Fréchet mean by $\Pi(V) = \{ \mu(V,p) : p\in\mathcal{S}^{k}\}$.

Here we establish some basic properties of $\Pi(V)$, while § 3.2 presents a more detailed analysis of $\Pi(V)$ within orthant interiors. First, the map $\mu$ is continuous and so $\Pi(V)$ is compact, since it is the continuous image of a compact set. Continuity of $\mu$ can be proved using the deterministic algorithm for calculating the weighted Fréchet mean given by Bačák (2014); the output of the algorithm depends continuously on the inputs $V$ and $p$. Secondly, the points $v_0,\ldots,v_k$ are contained in $\Pi(V)$, since $\mu(V,e_i)=v_i$ where $e_i$ denotes the $i$th standard basis vector in $\mathcal{S}^{k}$. Similarly, each geodesic $\Gamma(v_i,v_j)$ is contained in $\Pi(V)$, by taking $p$ to be a convex combination of $e_i$ and $e_j$. By the same argument, if $W$ is a nonempty subset of $V$, then $\Pi(V)$ contains $\Pi(W)$.

In Euclidean space the convex hull of $k+1$ points coincides with the locus of the Fréchet mean of the points. However, this is not the case in tree space, though $\Pi(V)$ is contained in the closure of the convex hull of $V$. This latter property follows because any point in $\Pi(V)$ can be approximated arbitrarily closely by performing a finite number of steps in the algorithm of Bačák (2014), as shown in § 2.2. Provided the algorithm is initialized with one of the points $v_0,\ldots,v_k$, each of these steps remains within the convex hull, and so the limit point is contained in the closure of the convex hull. Note that $\Pi(V)$ is itself generally not convex, so there may not be a unique closest point on $\Pi(V)$ to any given point $z$, although the minimum distance of $z$ from $\Pi(V)$ is well defined. By using $\Pi(V)$ as a principal component we have therefore lost the desirable property of uniqueness of projection.

Fréchet means in tree space exhibit a property called stickiness (Hotz et al., 2013). This essentially means that for fixed $V$ the map $\mu(V,\cdot):\mathcal{S}^{k}\rightarrow\mathcal{T}_{N}$ can fail to be injective. Specifically, depending on the points in $V$, there may exist open sets in $\mathcal{S}^{k}$ which all map to the same point in tree space. This has implications when we project data points onto $\Pi(V)$: given a data point $z$, the value of $p$ which minimizes $d\{z,\mu(V,p)\}^2$ might be nonunique, even if there is a unique closest point $x\in\Pi(V)$ to $z$.

### 3.2. Implicit equations for the locus of the Fréchet mean

The algebraic form of tree space geodesics described in § 2.1 can be used to derive implicit equations for the edge lengths of trees lying on the locus of the Fréchet mean $\Pi(V)$, and these equations are fundamental to establishing the dimension of $\Pi(V)$. For fixed $V=\{v_0, \ldots, v_k\}$, consider the objective function $\Omega:\mathcal{T}_{N}\times\mathcal{S}^{k}\rightarrow{\mathbb{R}}$ defined by

\begin{equation*}
\Omega(x,p) = \sum_{i=0}^k p_i\,d(x,v_i)^2\text{.}
\end{equation*}

Suppose we fix an orthant ${\mathcal{O}}_\tau$ for a fully resolved topology $\tau$. Let $x\in{\mathcal{O}}_\tau$ have edge lengths $x_j=|e_j|_x$ where $e_j\in{\mathcal{E}}({x})$$(j=1,\ldots,2N-2)$. Miller et al. (2015) showed that functions of the form $d(x,v_i)^2$ are continuously differentiable on ${\mathcal{O}}_\tau$ with respect to the edge lengths $x_j$. In order to minimize $\Omega$ we also assume that $x$ lies in a set

(4) \begin{equation*}\label{equ:msr} S = \bigcap_{i=0}^k S_{v_i}^\circ(\sigma_i,\tau) \end{equation*}

for some choice of supports $\sigma_0,\ldots,\sigma_k$. We call sets of this form mutual support regions with respect to $v_0,\ldots,v_k$. For each $i$ the sets $S_{v_i}^\circ(\sigma_i,\tau)$ are open and the union over possible choices $\sigma_i$ is dense in ${\mathcal{O}}_\tau$, as shown in § 2.1. Since the intersection of finitely many dense open sets is also dense, it follows that the union of sets of the form $S$ in (4) over all choices $\sigma_0,\ldots,\sigma_k$ is dense in ${\mathcal{O}}_\tau$. Each mutual support region is essentially a piece of tree space for which the combinatorics of the geodesics to $v_0,\ldots,v_k$ do not vary as a reference point moves around the region. An example of a decomposition of orthants into mutual support regions is given in § 3.4. Under this assumption on $x$, we can write down the algebraic form of $d(x,v_i)^2$ using (3), to give

\begin{align*}
\Omega(x,p) &= \|x\|^2 +\sum_{i=0}^kp_i \bigl( \|v_i\|^2+2\langle A_{xv_i}, B_{xv_i} \rangle-2\langle C_{xv_i}, D_{xv_i} \rangle \bigr)\nonumber\\
\end{align*}

so that

(5) \begin{align*} \frac{\partial\Omega}{\partial x_j} &= 2x_j+2\sum_{i=0}^kp_i\,\frac{\partial}{\partial x_j} \bigl( \langle A_{xv_i}, B_{xv_i} \rangle-\langle C_{xv_i}, D_{xv_i} \rangle \bigr)\text{.} \label{equ:diff_omega} \end{align*}

If the point $x\in S$ lies on the locus of the Fréchet mean $\Pi(V)$, then $\partial\Omega/\partial x_j=0$ for all $j$, and so we want to evaluate these derivatives to obtain implicit equations relating the edge lengths $x_j$ to the vector $p$.

Let $y$ be any of the trees $v_0,\ldots,v_k$. By definition, $\langle C_{xy}, D_{xy} \rangle = \sum_{e\in\mathcal{C}(x,y)}|e|_x|e|_y$, so

\begin{equation*}
\frac{\partial}{\partial x_j}\langle C_{xy}, D_{xy} \rangle = |e_j|_{y},
\end{equation*}

since $x_j$ is the length of split $e_j$. The derivative of $\langle C_{xy}, D_{xy} \rangle$ is therefore a constant. The term $\langle A_{xy}, B_{xy} \rangle$ has a more general functional dependence on $x_j$. By definition,

\begin{equation*}
\langle A_{xy}, B_{xy} \rangle = \sum_{l=1}^{\ell_{xy}}\|
A_{xy}^{(l)} \|_x\| B_{xy}^{(l)} \|_{y} =
\sum_{l=1}^{\ell_{xy}}\left( \sum_{e\in A_{xy}^{(l)} }|e|^2_x
\right)^{1/2} \left( \sum_{f\in B_{xy}^{(l)} }|f|^2_{y}
\right)^{1/2}\text{.}
\end{equation*}

For any edge $e_j\in\mathcal{C}(x,y)$ this expression does not depend on $x_j$, so the derivative is zero. When $e_j\in{\mathcal{E}}({x})\setminus\mathcal{C}(x,y)$, only the first term in brackets will depend on $x_j$. Since the sets $A_{xy}^{(l)}$ are disjoint, it must be the case that $e_j$ is contained in exactly one set, and we define $r_{ij}$ to be the index $l$ of that set when $y=v_i$. Then

\begin{equation*}
\frac{\partial}{\partial x_j}\langle A_{xv_i}, B_{xv_i} \rangle =
\|B_{xv_i}^{(r_{ij})}\|\frac{\partial}{\partial x_j}\left(
\sum_{e\in A_{xv_i}^{(r_{ij})}}| e |^2_x \right)^{1/2} =
x_j\frac{\|B_{xv_i}^{(r_{ij})}\|}{\|A_{xv_i}^{(r_{ij})}\|}\text{.}
\end{equation*}

In the case where $A_{xv_i}^{(r_{ij})}$ contains only $e_j$ and no other splits, we have $\|A_{xv_i}^{(r_{ij})}\|=x_j$, so the expression becomes $\partial\langle A_{xv_i}, B_{xv_i} \rangle / \partial x_j = \|B_{xv_i}^{(r_{ij})}\|$, which is also a constant. Substituting these expressions into (5) gives

(6) \begin{equation*}\label{equ:omega_deriv} \frac{\partial\Omega}{\partial x_j} = 2x_j + 2\sum_{i=0}^kp_i\,\left\{ x_j\frac{\|B_{xv_i}^{(r_{ij})}\|}{\|A_{xv_i}^{(r_{ij})}\|}(1-\mathcal{C}_{ij}) - |e_j|_{v_i}\mathcal{C}_{ij}\right\}, \end{equation*}

where $\mathcal{C}_{ij}=1$ if $e_j\in\mathcal{C}(x,v_i)$ and 0 otherwise.

We define $F: {\mathcal{O}}_\tau\times\mathcal{S}^{k}\rightarrow{\mathbb{R}}^{2N-2}$ by

(7) \begin{equation*}\label{equ:def_F} F(x, p) = \nabla_x\Omega(x,p)\text{.} \end{equation*}

Miller et al. (2015) showed that the function $d(x,y)^2$ for fixed $y$ is continuously differentiable on ${\mathcal{O}}_\tau$ with respect to $x\in{\mathcal{O}}_\tau$. Higher derivatives exist within each support region $S^\circ_y(\sigma,\tau)$. It follows that $F$ is continuously differentiable with respect to the edge lengths for all $x$ lying within the interior of mutual support regions, and that $F$ is continuous on ${\mathcal{O}}_\tau$. However, $F$ may not be differentiable on the boundary between mutual support regions. In § 3.3 we show that the matrix of second derivatives of $\Omega$ is positive definite on each mutual support region, and so every solution to $\nabla_x\Omega=0$ is a minimum. It follows that $\Pi(V)$ is locally the solution to $F(x,p)=0$.

The following lemma establishes conditions for $\Pi(V)$ to be a flat affine subspace within the mutual support region $S\subset{\mathcal{O}}_\tau$.

Lemma 1. *If the supports $\sigma_0,\ldots,\sigma_k$ are such that the geodesics $\Gamma(x,v_i)$ are simple for all $i=0,\ldots,k$, in the sense of Definition* 1, *then $\Pi(V)$ is an affine subspace of dimension $k$ or lower in $S = \bigcap_i S_{v_i}^\circ(\sigma_i,\tau)$.*

Proof. If all the geodesics $\Gamma(x,v_i)$ are simple for $x\in S$, then each set $A_{xv_i}^{(l)}$ contains exactly one split. Then (6) becomes

\begin{equation*}
\frac{\partial\Omega}{\partial x_j} = 2x_j +
2\sum_{i=0}^kp_i\alpha_{ij}
\end{equation*}

for some constants $\alpha_{ij}$. Solving $F(x,p)=0$ gives each edge length $x_j$ as a linear combination of $p_0,\ldots,p_k$, which establishes the result. Generically, $\Pi(V)$ is therefore locally a $k$-dimensional affine subspace of ${\mathcal{O}}_\tau$, but the dimension may be lower. Further discussion of the dimension is given in § 3.3. □

### 3.3. The dimension of the locus of the Fréchet mean

That $\Pi(V)$ has dimension $k$ in each mutual support region follows quickly from the form of $F$ in (7) through application of the implicit function theorem.

Lemma 2. *The matrix with elements $\partial F_j/\partial x_k$ is positive definite for all $x$ in mutual support region $S$.*

A proof of this lemma can be found in the Supplementary Material.

Theorem 1. *Within the mutual support region $S$, the locus of the Fréchet mean $\Pi(V)$ is a submanifold of dimension $k$ or lower. For generic selections of the points $v_0,\ldots, v_k$, the dimension is $k$.*

Proof. Application of the implicit function theorem to the map $F$ when $x\in S$ establishes that there is a locally defined function $g:\mathcal{S}^{k}\rightarrow S$ such that $F\{g(p),p\}=0$ and that the locus $\{g(p),p\}$ is a $k$-dimensional submanifold of $S\times\mathcal{S}^{k}$. In fact, the image $g(p)\subset S$ will be $k$-dimensional when $\nabla_pF$, the derivative of $F$ with respect to $p$, has rank $k$, which holds for generic selections of $V$ in tree space. This is analogous to considering the unique affine subspace containing $k+1$ given points in Euclidean space: generically the subspace has dimension $k$, but it can be lower. □

### 3.4. Explicit calculation

**Fig. 1.**
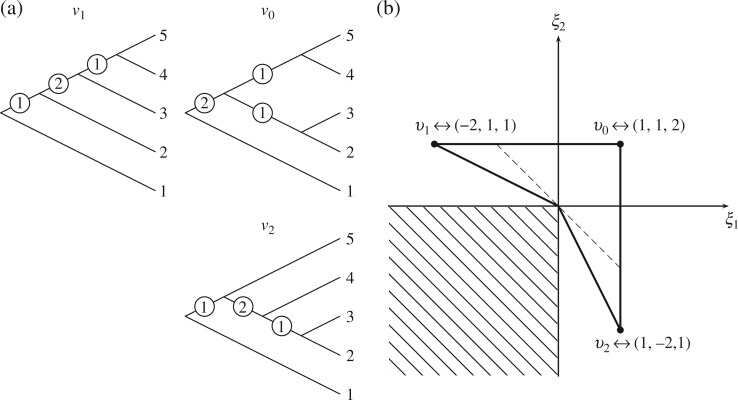
(a) Topologies for the trees $v_0,v_1,v_2$ of the example in § 3.4; the circled numbers are weights for internal edges. (b) Coordinates of the trees $v_0,v_1,v_2$ under the identification with orthants in ${\mathbb{R}}^3$; the $\xi_3$ axis points out of the page. The geodesics between $v_0,v_1,v_2$ are shown: $\Gamma(v_1,v_2)$ kinks around the origin; the dashed line is between points $(-1,1,4/3)$ and $(1,-1,4/3)$ on $\Gamma(v_0,v_1)$ and $\Gamma(v_0,v_2)$, respectively; the lower left quadrant does not correspond to any tree topology, and is not a part of the space.

In this subsection we construct an explicit example of the locus of the Fréchet mean for three points in $\mathcal{T}_{5}$. This example helps to demonstrate the nature of geodesics in tree space, the derivation of the implicit equations for $\Pi(V)$, the relationship with the convex hull, and other geometrical features. We start by fixing $v_0,v_1$ and $v_2$ to have the topologies and edge lengths shown in Fig. 1(a). We will ignore the pendant edge lengths, and so the orthants containing these trees can be identified with three orthants in ${\mathbb{R}}^3$ equipped with standard coordinates $\xi_1,\xi_2,\xi_3$. There are five splits contained in these trees, excluding the pendant splits; they will be written as $\{0,1\}$, $\{2,3\}$, $\{4,5\}$, $\{3,4,5\}$ and $\{2,3,4\}$ by neglecting the complements in $X=\{0,1,\ldots,N\}$. We then let $x(\{0,1\})$ denote the length associated with split $\{0,1\}$ in tree $x$, for example. Under the identification with ${\mathbb{R}}^3$ we have

\begin{align*}
\xi_1&=x(\{2,3\})\quad (\{2,3\}\in x),\qquad \xi_1=-x(\{3,4,5\})\quad(\{3,4,5\}\in x),\\
\xi_2&=x(\{4,5\})\quad(\{4,5\}\in x),\qquad
\xi_2=-x(\{2,3,4\})\quad(\{2,3,4\}\in x)
\end{align*}

and $\xi_3 = x(\{0,1\})$. Figure 1(b) shows the location of trees $v_0,v_1,v_2$ under this identification. The orthant $\xi_1<0, \xi_2<0, \xi_3>0$ does not correspond to a valid tree topology as $\{3,4,5\}$ is not compatible with $\{2,3,4\}$. At each codimension-$1$ face between the orthants shown there is in fact a third orthant in $\mathcal{T}_{5}$ glued at the same boundary, but these orthants do not play a role in this example.

In Fig. 1(b) it can be seen that the geodesics $\Gamma(v_0,v_1)$ and $\Gamma(v_0,v_2)$ are straight-line segments under the identification with ${\mathbb{R}}^3$, while the geodesic $\Gamma(v_1,v_2)$ kinks at a codimension-$2$ face. This behaviour is typical of geodesics in $\mathcal{T}_{N}$: they are straight-line segments within each orthant but can contain kinks at the boundaries between orthants. Figure 1(b) also shows how the convex hull of $v_0,v_1,v_2$ has dimension 3. The dashed line shows the geodesic between points $(-1,1,4/3)$ and $(1,-1,4/3)$ on $\Gamma(v_0,v_1)$ and $\Gamma(v_0,v_2)$, respectively. The convex hull therefore contains the points $(0,0,1)$ and $(0,0,4/3)$, so there are four points which are not coplanar within each orthant of the convex hull.

**Fig. 2.**
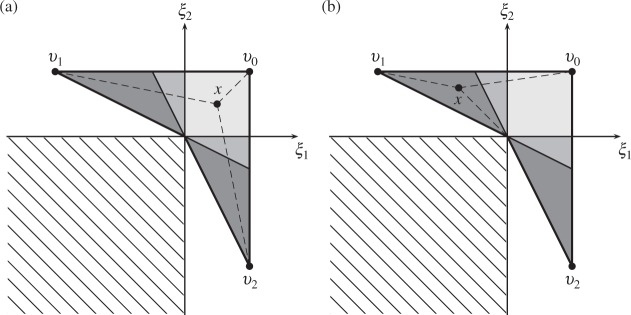
Decomposition of the locus of the Fréchet mean into mutual support regions. There are five such regions, represented by shading: two mutual support regions are dark grey, and two are mid-grey. The dashed lines show the geodesics between a point $x$ and the points $v_0,v_1,v_2$: (a) when $x$ is contained in the light grey mutual support region, none of the geodesics $\Gamma(x,v_i)$ hit codimension-$2$ orthant faces, so Lemma 1 shows that $\Pi(V)$ is planar within the region; the same applies to the two mutual support regions shaded mid-grey; (b) when $x$ is contained in one of the dark grey shaded regions, then $\Gamma(x,v_2)$ is not simple as it intersects a codimension-$2$ boundary, so the part of $\Pi(V)$ lying within this region is not planar.

Figure 2 shows the decomposition of the orthants into mutual support regions for $v_0,v_1$ and $v_2$. There are five regions in total, and the geodesics $\Gamma(x,v_i)$ are simple for all $i=0,1,2$ when $x$ is contained in three of the regions. Lemma 1 shows that $\Pi(V)$ is therefore planar in those regions with equation

\begin{equation*}
\xi = (p_0-2p_1+p_2, \,p_0+p_1-2p_0, \,1+p_0)\text{.}
\end{equation*}

We can also explicitly calculate equations for $\Pi(V)$ in the mutual support region contained in $2\xi_1+\xi_2<0$ and shown in dark grey at the top-left of each panel in Fig. 2. For $x$ contained in this region, the squared distances to the vertices are

\begin{align*}
d(x,v_0)^2 &= (1-\xi_1)^2 + (1-\xi_2)^2 + (2-\xi_3)^2,\\
d(x,v_1)^2 &= (2+\xi_1)^2 + (1-\xi_2)^2 + (1-\xi_3)^2,\\
d(x,v_2)^2 &= \bigl\{ 5^{1/2}+(\xi_1^2+\xi_2^2)^{1/2} \bigr\}^2 +
(1-\xi_3)^2,
\end{align*}

where $x$ has coordinates $\xi_1,\xi_2,\xi_3$. These can be used to write down an equation for $\Omega(x,p)$, and then (6) becomes

\begin{equation*}
\nabla_\xi\Omega = \left(
2\xi_1+2\frac{p_2\xi_15^{1/2}}{(\xi_1^2+\xi_2^2)^{1/2}}+4p_1-2p_0
,\:
2\xi_2+2\frac{p_2\xi_15^{1/2}}{(\xi_1^2+\xi_2^2)^{1/2}}-2p_1-2p_0
,\: 2p_0+2-2\xi_3 \right)\text{.}
\end{equation*}

Then $\nabla_\xi\Omega=0$ can be solved to give

**Fig. 3.**
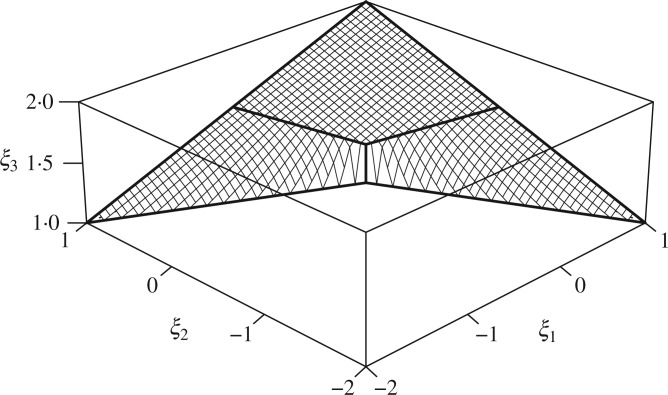
Perspective view of $\Pi(V)$ for the example in § 3.4. The locus of the Fréchet mean is a two-dimensional surface which resembles a rubber sheet pulled taut between the corners.

\begin{equation*}
\xi = \left( p_0-2p_1+p_2 \bigl[5\big/\bigl\{ 1+f(p)^2
\bigr\}\bigr]^{1/2} ,\; p_0+p_1-p_2\bigl[5\big/\bigl\{ 1+f(p)^{-2}
\bigr\}\bigr]^{1/2} ,\; p_0+1 \right)
\end{equation*}

whenever $p_0<2p_1$, where $f(p) = (p_0+p_1)/(p_0-2p_1)$. The resulting surface is shown in Fig. 3, from which we can see that $\Pi(V)$ forms a nonconvex two-dimensional surface that is contained within the convex hull.

## 4. Projection onto the locus of the Fréchet mean and principal component analysis

### 4.1. Projection

In order to use the surface $\Pi(V)$ as a principal component, we need to be able to project data onto $\Pi(V)$. Let $z\in\mathcal{T}_{N}$ denote a data point and fix $V=\{v_0,\ldots,v_k\}$. A projection of $z$ onto $\Pi(V)$ is a point which minimizes $d\{z,\Pi(V)\}$. This point may not be unique as $\Pi(V)$ is not convex. A naive algorithm to find a projection is to perform an exhaustive search, as described in Algorithm 2.

Algorithm 2. Exhaustive search to project $z$ onto $\Pi(V)$.

Construct a lattice of points $L\subset\mathcal{S}^{k}$. For $k=2$ this is a triangular lattice.

For each point $p\in L$ use a standard algorithm to compute $\mu(V,p)$.

Find $p\in L$ which minimizes $d\{z,\mu(V,p)\}$.

We implemented this algorithm for $k=2$ and used the algorithm of Bačák (2014) in the second step to compute Fréchet means. Algorithm 2 is computationally very expensive, since the resolution of the lattice $L$ needs to be quite fine in order to obtain accurate results. Consequently we use the exhaustive search algorithm only as a benchmark for assessing other methods.

We would like a more efficient algorithm defined entirely in terms of the geodesic geometry, since any reliance on local differentiable structure is likely to be problematic at orthant boundaries. We propose Algorithm 3, which we call the geometric projection algorithm.

Algorithm 3. Geometric projection algorithm to project $z$ onto $\Pi(V)$.

Fix an initial estimate $\mu_0$ of the projection of $z$, let $p=(0,\ldots,0)$, and set $i=0$.

Repeat:

 Construct $\Gamma(\mu_i,v_j)$ for $j=0,\ldots,k$.

 For $j=0,\ldots, k$ let $y_{i,j}$ be the point a proportion $s_i=1/(i+2)$ along $\Gamma(\mu_i,v_j)$.

 Find $r\in\{0,\ldots,k\}$ which minimizes $d(z,y_{i,r})$.

 Set $\mu_{i+1}\leftarrow y_{i,r}$ and $p\leftarrow ip/(i+1)+e_r/(i+1)$, where $e_r$ is the $r$th standard basis vector

  in $\mathcal{S}^{k}$.

 Set $i\leftarrow i+1$.

Until the sequence $\mu_0,\mu_1,\ldots$ converges.

Algorithm 3 is a modification of Sturm’s algorithm for computing the Fréchet mean of $V$, Algorithm 1. At each step of Sturm’s algorithm, one of the points $y_{i,j}$ is used as the new estimate $\mu_{i+1}$, and the point $y_{i,j}$ is sampled according to a fixed probability vector $p$. Here, the new estimate for the projection, $\mu_{i+1}$, is again chosen from $y_{i,0},\ldots,y_{i,k}$ but is selected to greedily minimize the distance from $z$. The vector $p\in\mathcal{S}^{k}$ estimates the weight vector associated with the projected point: at iteration $i$, $\:i\times p$ is a vector with integer entries which counts the number of times the algorithm has moved the estimate of the projection towards each vertex in $V$. The computational cost of the algorithm is similar to that for computing a single Fréchet mean using the Sturm algorithm. For $k=2$ the initial point $\mu_0$ is sampled uniformly from the perimeter of $\Pi(V)$. Convergence is tested as follows: at iteration $i$ it is determined whether $d(\mu_s,\mu_t)<\epsilon$ for all $s,t\in\{i-m,\ldots,i\}$, where $\epsilon>0$ and $m$ are fixed; if that is the case, then the algorithm terminates. The output from the algorithm after $I$ iterations is an estimate $\mu_I$ of the projection of $z$ and a vector $p\in\mathcal{S}^{k}$.

The geometric projection algorithm is presented here without a proof of convergence and without further theoretical study of its properties. Instead we rely on a simulation study in the next subsection to assess its effectiveness.

### 4.2. Simulations

We ran simulations designed to demonstrate that, specifically in the case of $k=2$, Algorithm 3 converges to a tree on $\Pi(V)$ which minimizes $d\{z,\Pi(V)\}$. For each iteration of the simulation, a random species tree $u$ with $N=6$ taxa was generated under the Kingman (1982) coalescent. Three trees $v_0,v_1,v_2$ and a fourth test tree $z$ were then generated under a coalescent model constrained to be contained within the tree $u$, and thus corresponded to gene trees coming from the underlying species tree $u$. Maddison (1997) describes in detail the relationship between species trees and gene trees. The DendroPy library (Sukumaran & Holder, 2010) was used to generate these trees. The test tree $z$ was then projected onto $\Pi(V)$ for $V=\{v_0,v_1,v_2\}$ using the exhaustive search algorithm and the geometric projection algorithm. All calculations were carried out ignoring pendant edges. This particular simulation scheme was chosen in order to generate a variety of different geometrical configurations for the points $v_0,v_1,v_2$ and $z$, as well as being biologically reasonable. If the trees were sampled with topologies chosen independently uniformly at random, for example, the simulation procedure would only have explored instances of $\Pi(V)$ with widely differing vertices.

The results obtained from the two algorithms were compared in two ways. First, the distances from the data tree to the projected trees obtained with the two algorithms were computed and checked to ensure that the projection algorithm yielded a distance less than or equal to the exhaustive search. Second, the distance between the tree from geometric projection and the tree from exhaustive search was checked to ensure that the two trees were close together. For the second check we considered any distance greater than 1% of the total internal length of the data tree to be a failure.

In a run of 10 000 replications of this procedure, 95$\cdot$7% of the replications passed the two tests. However, even the set of failing replications produced a projection result that was quite close to the exhaustive search result. Among the 435 failing replications, the perpendicular distance for the projection was an average of 3$\cdot$7% greater than the perpendicular distance of the exhaustive search, and the distance between the two results was an average of 4$\cdot$7% of the total internal length of the data tree.

We believe that the failing results are attributable to the projection algorithm becoming trapped in local minima of the perpendicular distance. Starting the algorithm from several locations and comparing the results would help to mitigate this problem. However, for the present purpose of fitting higher principal components to a collection of data trees, we believe these small deviations from the exhaustive search solution are an acceptable trade for the increase in computational speed.

### 4.3. Stochastic optimization for principal component analysis

Given data $Z=\{z_1,\ldots,z_n\}$, our objective is to find $V=\{v_0,\ldots,v_k\}$ that minimizes the sum of squared projected distances $D^2_Z\{\Pi(V)\}$. We henceforth restrict ourselves to the case $k=2$. The geometric projection algorithm is used to compute $D^2_Z\{\Pi(V)\}$ given $V$, at least approximately, so we must now consider how to search over the possible configurations of the vertices $V$. We adopt a stochastic optimization approach, Algorithm 4 below, which is similar to that used for fitting principal geodesics in Nye (2014). We assume that we have available a set of proposals $M_1,\ldots,M_m$, each of which is a map from $\mathcal{T}_{N}$ to the set of distributions on $\mathcal{T}_{N}$. In particular, given any tree $x$, each $M_i(x)$ is assumed to be a distribution on $\mathcal{T}_{N}$ from which we can easily sample.

Algorithm 4. Stochastic optimization algorithm to fit $\Pi(V)$ to $Z$.

Fix an initial set $V=\{v_0,v_1,v_2\}$ and compute $D^2_Z\{\Pi(V)\}$.

Repeat:

  For $i=0,1,2$:

  For $j=1,\ldots,m$:

    Sample a tree $w$ from $M_j(v_i)$.

    Let $V'$ be the set $V$ but with $w$ replacing $v_i$.

    Compute $D^2_Z\{\Pi(V')\}$ using the geometric projection algorithm.

    If $D^2_Z\{\Pi(V')\}<D^2_Z\{\Pi(V)\}$ set $V\leftarrow V'$.

Until convergence.

The optimization algorithm attempts to minimize $D^2_Z\{\Pi(V)\}$ by stochastically varying one point $v\in V$ at a time using the proposals $M_i(v)$. The algorithm is greedy: whenever a configuration $V'$ improves upon the current configuration $V$ we replace $V$ with $V'$. Convergence is assessed by considering the relative change in $D^2_Z\{\Pi(V)\}$ over a certain fixed number of iterations. If this is less than some proportion then the algorithm terminates. We used three different types of proposal. The first samples a tree uniformly at random with replacement from the dataset $Z$. The second type is a refinement of the first: given a tree $x$ it similarly samples a tree $z$ uniformly at random with replacement from the dataset $Z$; then the geodesic $\Gamma(x,z)$ is computed, and a beta distribution is used to sample a tree some proportion of the distance along $\Gamma(x,z)$. The third type of proposal is a random walk starting from $x$, as described in Nye (2014). The random walk proposals can have different numbers of steps and step sizes. The algorithm is not guaranteed to find a global optimum, and it can become stuck in local minima, so the algorithm must be run with different starting points for each dataset, and then compare the results from each run.

Two statistics can be used to summarize the fit of $\Pi(V)$ to a dataset $Z$: the sum of squared projected distances $D^2_Z\{\Pi(V)\}$ and a non-Euclidean proportion of variance statistic, denoted by $r^2$. If the projection of each data point $z$ onto $\Pi(V)$ is denoted by $\pi(z_i)$ and $\bar{\pi}$ denotes the Fréchet mean of $\pi(z_1),\ldots,\pi(z_n)$, then

\begin{equation*}
r^2 = \frac{ \sum_{i=1}^n d\{z_i, \pi(z_i)\}^2 }{ \sum_{i=1}^n d\{z_i, \pi(z_i)\}^2 +\sum_{i=1}^n d\{\bar{\pi}, \pi(z_i)\}^2
}\text{.}
\end{equation*}

The denominator in this expression varies with $\Pi(V)$ since Pythagoras’ theorem does not hold in tree space. Unlike $D^2_Z\{\Pi(V)\}$, the $r^2$ statistic is quite sensitive to small changes in $V$, but it can be interpreted broadly as the proportion of variance explained by $\Pi(V)$.

To assess the performance of the algorithm we conducted a small simulation study. Eight datasets of 100 trees containing $N=10$ taxa were generated in the following way. For each dataset a tree topology was sampled from a coalescent process, and each edge length was sampled from a gamma distribution with shape $\alpha=2$ and rate $\beta=20$, to give a tree $w_0$. Two trees $w_1$ and $w_2$ were then obtained by applying random topological operations to $w_0$. In four of the datasets, $w_1$ and $w_2$ were obtained by performing nearest-neighbour interchange operations, while in the other four datasets subtree prune and regraft operations were used. Then, to construct each dataset given $W=\{w_0,w_1,w_2\}$, 100 points were sampled from a Dirichlet distribution on $\mathcal{S}^{2}$ with parameter $(4,4,4)$, and the corresponding points on $\Pi(W)$ were found using the Bačák algorithm. Each point was then perturbed by using a random walk, so that each dataset resembled a cloud of points around the surface $\Pi(W)$. The step size of the random walk was tuned to produce datasets classified as having either low or high dispersion. Table 1 summarizes the datasets used and the simulation results. The stochastic optimization algorithm performs well in every scenario.

**Table 1.** Simulations to assess the stochastic optimization algorithm: the leftmost column describes the number and type of topological operation used to obtain $w_1$ and $w_2$ from $w_0$ for each dataset; in each scenario, two datasets were generated by perturbing points on $\Pi(W)$ via random walks, with low and high dispersions. Shown are the fitted values $D^2_Z\{\Pi(V)\}$ computed with the geometric projection algorithm, with reference values $D^2_Z\{\Pi(W)\}$ in parentheses, computed with the exhaustive projection algorithm, together with the non-Euclidean $r^2$ statistic, with reference values in parentheses

	Low dispersion		High dispersion	
Topological scenario	$D^2_Z$	$r^2\,({\%})$	$D^2_Z$	$r^2\,({\%})$
$2 \times$ nearest-neighbour interchange	$0{\cdot}28\;(0{\cdot}27)$	$41\;(50)$	$2{\cdot}7\;(2{\cdot}7)$	$18\;(18)$
$4 \times$ nearest-neighbour interchange	$0{\cdot}31\;(0{\cdot}30)$	$61\;(66)$	$2{\cdot}6\;(2{\cdot}9)$	$27\;(20)$
$2 \times$ subtree prune and regraft	$0{\cdot}26\;(0{\cdot}25)$	$59\;(62)$	$2{\cdot}1\;(2{\cdot}4)$	$29\;(21)$
$4 \times$ subtree prune and regraft	$0{\cdot}27\;(0{\cdot}28)$	$54\;(48)$	$2{\cdot}4\;(2{\cdot}8)$	$24\;(22)$

## 5. Results

### 5.1. Coelacanths genome and transcriptome data

We applied our method to the dataset comprising 1290 nuclear genes encoding 690 838 amino acid residues obtained from genome and transcriptome data by Liang et al. (2013). Over the past few decades researchers have worked on the phylogenetic relations between coelacanths, lungfishes and tetrapods, but controversy remains despite several studies (Hedges, 2009). Most morphological and palaeontological studies support the hypothesis that lungfishes are closer to tetrapods than they are to coelacanths. However, some research supports alternative hypotheses: that coelacanths are closer to tetrapods; that coelacanths and lungfish are closest; or that tetrapods, lungfishes and coelacanths cannot be resolved. Liang et al. (2013) present these four hypotheses in their Fig. 1, Trees 1–4, respectively.

We reconstructed gene trees using the R (R Development Core Team, 2017) package Phangorn (Schliep, 2011), with each gene tree estimated using maximum likelihood under the Le & Gascuel (2008) model. The dataset consisted of 1290 gene alignments for 10 species: lungfish, *Protopterus annectens*, and coelacanth, *Latimeria chalumnae*; three tetrapods, frog, *Xenopus tropicalis*, chicken, *Gallus gallus*, and human, *Homo sapiens*; two ray-finned fish, *Danio rerio* and *Takifugu rubripes*; and three cartilaginous fish included as an out-group, *Scyliorhinus canicula*, *Leucoraja erinacea* and *Callorhinchus milii*.

**Fig. 4.**
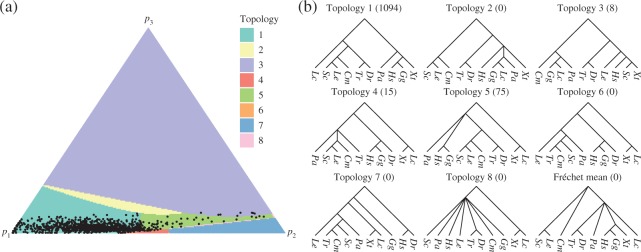
The second principal component computed from the lungfish dataset: (a) the simplex shaded according to the topology of the corresponding points on $\Pi(V)$, with the projections of the data points also displayed; (b) topologies of trees on $\Pi(V)$. Species abbreviations are based on the binary nomenclature: lungfish, *Pa*; coelacanth, *Lc*; frog *Xt*; chicken, *Gg*; human, *Hs*; ray-finned fish, *Dr* and *Tr*; cartilaginous fish, *Sc*, *Le* and *Cm*. The number of data points projecting to each topology is displayed in brackets.

Analysis was performed ignoring pendant edge lengths. A total of 97 outlying trees were removed using KDETrees (Weyenberg et al., 2016), so that 1193 gene trees remained. The Fréchet mean was computed using the Bačák algorithm and its topology is shown in Fig. 4. The mean tree does not resolve whether coelacanth or lungfish is the closest relative of the tetrapods. The sum of squared distances of the data points to the Fréchet mean was 19$\cdot$7. A principal geodesic was constructed using the algorithm from Nye (2014): the sum of squared projected distances was 9$\cdot$53 and the non-Euclidean $r^2$ statistic was 51$\cdot$4%. Traversing the principal geodesic gives trees with the same topology as the Fréchet mean that contract down to a star tree at one end of the geodesic and expand in size at the other end. This shows that the principal source of variation in the dataset is the overall scale of the gene trees or, in other words, the total amount of evolutionary divergence for each gene.

Figure 4 illustrates the second principal component. The sum of squared projected distances was 7$\cdot$29 and the non-Euclidean $r^2$ statistic was 61$\cdot$8%. This represents a relatively small increase in the proportion of variance in relation to the principal geodesic. Three runs of Algorithm 4 were performed to construct the second principal component. The results obtained had very similar summary statistics, but the topologies displayed on the surfaces were more variable, so Fig. 4 is a representative choice. Although the projected points are clustered towards the bottom of the simplex in the figure, the full simplex was drawn to show all the different topological regions. Of the 1193 gene trees, 1094 projected to points with topology 1, which supports lungfish being the closest relative of the tetrapods. From the remaining projected data points, 75 have topology 5, placing both lungfish and coelacanth in a clade with the tetrapods. The topologies 3, 4, 6 and 7 have biologically implausible relationships. However, the projected data points lying outside topology 1 all lie close to the boundary of their respective orthants, having at least one edge length less than 0$\cdot$0005. For example, the projected data points with topology 3 have very short edge lengths for the biologically implausible clades, such as the grouping of *X. tropicalis* with *S. canicula*, and so lie close to trees with more plausible topologies.

Overall, the second principal component suggests that the data support topology 1, with lungfish as the closest relative of tetrapods, and that most of the variation within the data comes from edge length variation within that topology rather than from conflicting topologies. Although the estimates are subject to random variation, it is interesting that the Fréchet mean and principal geodesic did not exhibit topology 1, while the second principal component suggests a solution to the controversial relationship between coelacanth, lungfish and tetrapods. The exhaustive projection algorithm was used to project the data onto the surface $\Pi(V)$ produced by Algorithm 4, in order to compare with the results obtained by geometric projection. The sum of squared distances between the projected trees obtained with the two different algorithms was 0$\cdot$004, a small fraction of the sum of squared projected distances 7$\cdot$29 for $\Pi(V)$.

### 5.2. Apicomplexa

We also applied our method to a set of trees constructed from 268 orthologous sequences from eight species of protozoa in the Apicomplexa phylum, previously presented by Kuo et al. (2008). The same dataset was also analysed by Weyenberg et al. (2016), and more details are given in that paper, such as the gene sequences used to infer each tree. The phylum Apicomplexa contains many important protozoan pathogens (Levine, 1988), including the mosquito-transmitted *Plasmodium* species, the causative agent of malaria; *T. gondii*, which is one of the most prevalent zoonotic pathogens worldwide; and the water-borne pathogen *Cryptosporidium* species. Several members of the Apicomplexa also cause significant morbidity and mortality in both wildlife and domestic animals. These include the *Theileria* and *Babesia* species, which are tick-borne haemoprotozoan ungulate pathogens, and several species of *Eimeria*, which are enteric parasites that are particularly detrimental to the poultry industry. Because of their medical and veterinary importance, whole-genome sequencing projects have been completed for multiple prominent members of the Apicomplexa. We removed 16 outlier trees previously identified by Weyenberg et al. (2016) before fitting principal components.

**Fig. 5.**
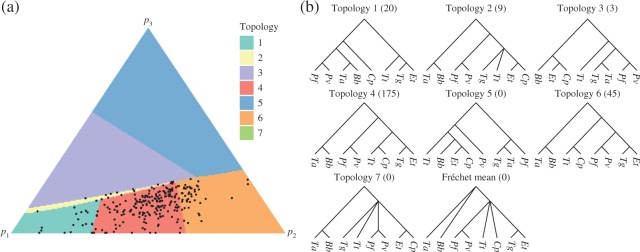
The second principal component computed from the Apicomplexa dataset: (a) the simplex shaded according to the topology of the corresponding points on $\Pi(V)$, with the projections of the data points also displayed; (b) topologies of trees on $\Pi(V)$. Species abbreviations are based on the species’ binary nomenclature. The number of data points projecting to each topology is displayed in brackets.

The trees were analysed ignoring pendant edges. The Fréchet mean was computed using the Bačák algorithm: the corresponding tree topology was unresolved, and is shown in Fig. 5. The sum of squared distances from the mean to the data points was 24$\cdot$6. The principal geodesic was estimated using the algorithm from Nye (2014). The principal geodesic has a non-Euclidean $r^2$ statistic of 40%, and the sum of squared projected distances was 14$\cdot$2. The principal geodesic displays two main effects. First, the edges leading to the *P. vivax* and *P. falciparum* clade, the *E. tenella* and *T. gondii* clade, and the *B. bovis* and *T. annulata* clade vary substantially in length. The second is a topological rearrangement whereby the clade containing *P. vivax* and *P. falciparum* paired with *E. tenella* and *T. gondii* is replaced with a clade containing *P. vivax* and *P. falciparum* paired with *B. bovis* and *T. annulata*. However, the second effect involved very short internal edges, so that along its length, the trees on the principal geodesic resembled the mean tree shown in Fig. 5 but with different overall scale. The principal geodesic therefore reflects variation in the scale of the tree.

Figure 5 illustrates the second principal component, with the simplex shaded according to the corresponding tree topology on $\Pi(V)$. Three separate runs of Algorithm 4 converged to give similar results. The summary statistics for the second principal component are: sum of squared projected distances 10$\cdot$3; non-Euclidean $r^2$ statistic 56%. While these summary statistics were consistent between runs, the set of topologies displayed on $\Pi(V)$ was subject to more variation, so Fig. 5 is a representative choice, although topologies 1, 4 and 6 were present in all runs. The results show how the second principal component is able to tease out more from the data than the variation in overall scale captured by the principal geodesic. Topology 4 is congruent with the generally accepted phylogeny of taxa within the Apicomplexa and is a resolution of the Fréchet mean tree: *T. annulata* and *B. bovis* group together; the two *Plasmodium* species group together; *C. parvum* is the deepest rooting apicomplexan; and *P. vivax*, *P. falciparum*, *T. annulata* and *B. bovis* are monophyletic. The latter group are all haemosporidians or blood parasites.

Figure 5 shows that the second principal component corresponds to variation in topology consisting of nearest-neighbour interchange operations that transform topology 4 into topologies 1 and 6. None of the projected trees have topology 5, although this is the topology of one of the vertices of $\Pi(V)$. This topology appears to be present in order for $\Pi(V)$ to be positioned in such a way as to capture the other topologies. Topology 2 shows evidence of stickiness, as discussed in § 3.1. Although the topology is unresolved, so that the coloured triangle lies in a codimension-$1$ region of tree space, it occupies the nonzero area on the simplex. As for the lungfish, the exhaustive and geometric projection algorithms were compared on the surface $\Pi(V)$ produced by Algorithm 4. The distances between the projected points obtained with the two algorithms were very small compared to the distances of the data points from $\Pi(V)$: the sum of squared distances between pairs of projected points was $3{\cdot}91\times 10^{-4}$.

## 6. Discussion

This paper presents three main innovations: (i) use of the locus of the Fréchet mean $\Pi(V)$ as an analogue of a principal component in tree space; (ii) proof that $\Pi(V)$ has the desired dimension; and (iii) the geometric projection algorithm for projecting data onto $\Pi(V)$. The locus of the Fréchet mean was first proposed as a geometric object for principal component analysis in tree space in a 2015 University of Kentucky PhD thesis by G. Weyenberg. Pennec (2015) made a similar proposal for an analogue of principal component analysis in Riemannian manifolds and other geodesic metric spaces, called barycentric subspace analysis. The barycentric subspaces of Pennec correspond exactly to the surfaces $\Pi(V)$ considered in this paper, except that the weights $p_0,\ldots,p_k$ are not constrained to lie in the simplex and can be negative. Pennec’s approach, however, is principally based in the context of a Riemannian manifold rather than in tree space, though he points out the potential for generalization. There are substantial differences between barycentric subspace analysis and the method presented in this paper. In particular, a key aim of barycentric subspace analysis is to produce nested principal components, $\Pi_0\subset\Pi_1\subset\Pi_2\subset\cdots$, while we do not have that restriction here. The nesting is achieved by either adding or removing points from $V$ in order to obtain, respectively, a higher- or lower-order nested principal component. This is also possible in the context of our analysis, but the $k$th principal component would in each case form part of the boundary of the $(k+1)$th principal component. This is undesirable as it leads to poorly fitting principal components. For example, suppose that the second principal component is constructed by adding an extra vertex to the principal geodesic; many data points would project onto the edge of the second principal component corresponding to the principal geodesic rather than being distributed over the interior of the surface. Similar problems arise if the analysis is performed by removing points from $V$ sequentially. These problems do not arise with Pennec’s methodology, because the weights $p_0,\ldots,p_k$ are not restricted to the simplex, so a nested principal component can lie in the interior of higher-order components. In contrast, the existing algorithms for computing the Fréchet mean in tree space and our algorithm for projection onto $\Pi(V)$ all require the weights $p_0,\ldots,p_k$ to lie in the simplex, and this motivated the decision to consider principal components which are not nested in this paper. If these algorithms could be adapted to allow negative values for the weights, then a nested principal component analysis would be possible in tree space.

Our analysis has been restricted to datasets with relatively few taxa and to the construction of the first and second principal components. The algorithms presented in this paper scale linearly with respect to the number of data points $n$, but run in polynomial time with respect to the number of taxa $N$. However, by partitioning the dataset for the geometric projection algorithm, parallel computer architectures can be employed and the speed-up is approximately proportional to the number of processors used. While the geometric projection algorithm runs relatively quickly, the calculations involved in searching for the optimal set of vertices $V$ can be very substantial. The experimental datasets in § 5 took between one and three days to analyse, running on four processors each. For higher-order components, $k>2$, this computational burden will increase, and it is likely that finding a global minimum for $D^2_Z\{\Pi(V)\}$ will be more difficult. While the method presented in this paper generalizes to arbitrary $k$, including the geometric projection algorithm, computational issues limited our analysis to $k\leq 2$. However, fitting a principal component $\Pi(V)$ with $k=3$ would give an upper bound on $D^2_Z\{\Pi(V)\}$ even if a global minimum were not found, and hence an approximate lower bound on the non-Euclidean $r^2$ statistic. Consequently, even a poorly fit principal component with $k=3$ might give some indication of the additional variance explained by higher-order components.

Uncertainty in estimated principal components could be assessed by bootstrap methods; for example, one can generate replicate datasets by resampling the data $z_1,\ldots,z_n$ and constructing principal components for each replicate. An alternative bootstrap procedure involves estimating a principal component $\Pi(V)$ for $z_1,\ldots,z_n$ and then generating replicate datasets by randomly perturbing the projection of each point $z_i$ onto $\Pi(V)$ using a random walk, in a similar way to the simulations in § 4.3. However, both these approaches are highly computationally expensive, and would only be feasible for relatively small datasets. Obtaining analytical results about uncertainty, such as proving validity of the bootstrap procedure or establishing confidence regions for principal components, would involve development of asymptotic theory on the space of configurations of the vertices $V$, and this lies well beyond existing probability theory on tree space (Barden et al., 2013).

The figures in § 5 demonstrate the potential for creating visualizations of the data which reveal meaningful biological structure. The pattern of projected points obtained for the experimental datasets we considered were very similar to results obtained via multi-dimensional scaling. However, multi-dimensional scaling is not capable of revealing the features of the dataset that cause the observed variation. More information could be included in the graphical representation of our results, such as the distance of the data points from their projections, information about the principal geodesic, and the proximity of points to orthant boundaries.

Our software for finding principal components in tree space is available to download from http://www.mas.ncl.ac.uk/~ntmwn/geophytterplus/. The datasets analysed in this paper are also available from that website. An optional R package used to produce the figures in this article can be obtained from https://github.com/grady/geophyttertools.

We presented Algorithm 3, the geometric projection algorithm, without a proof of convergence, and we used simulation to assess its accuracy. The algorithm is attractive in that it is defined entirely in terms of the geodesic structure on tree space, so it could be used on any geodesic metric space, including Riemannian manifolds. The algorithm clearly deserves further investigation, and we intend to study its properties in future work.

## Supplementary Material

Supplementary Data 1

Supplementary Data 2

## Supplementary material

Supplementary material available at *Biometrika* online includes the proof of Lemma 2 and the geophytter+ software, which implements the algorithms described in this paper.
