# Supplementary material for: Principal component analysis and the locus of the Fréchet mean in the space of phylogenetic trees
Source: Biometrika. 2017 Sep 27;104(4):901–22. doi: 10.1093/biomet/asx047 (PMC5793493; doi:10.1093/biomet/asx047)
Supplement: Supplementary Data 1 [file asx047_supp.pdf]

Supplementary material for Principal Component Analysis and the Locus of the Fréchet Mean in the Space of Phylogenetic Trees

This supplement gives a proof of the following lemma from the main body of the paper.

Lemma 2. *The matrix with elements $\partial F_j/\partial x_k$ is positive definite for all x in mutual support region S.*

Proof. Using equation (6) we have

$$\frac{\partial F_j}{\partial x_k} = 2\delta_{jk} + 2\sum_{i=0}^{k} p_i Q_{jk}^{(i)}$$

where the matrix $Q^{(i)}$ has elements

$$Q_{jk}^{(i)} = \frac{\partial}{\partial_k}\left(x_j \frac{\|B_{xv_i}^{(r_{ij})}\|}{\|A_{xv_i}^{(r_{ij})}\|}(1-\mathcal{C}_{ij}) - |e_j|_{v_i}\mathcal{C}_{ij}\right).$$

We start by assuming $\mathcal{C}_{ij} = 0$ for all i, j (so that $e_i \notin \mathcal{C}(x, v_i)$ for all i, j), and drop this assumption later. Then

$$\begin{aligned} Q_{jk}^{(i)} &= \delta_{jk}\frac{\|B_{xv_i}^{(r_{ij})}\|}{\|A_{xv_i}^{(r_{ij})}\|} - x_j \frac{\|B_{xv_i}^{(r_{ij})}\|}{\|A_{xv_i}^{(r_{ij})}\|^2}\frac{\partial}{\partial x_j}\|A_{xv_i}^{(r_{ij})}\| \\ &= \delta_{jk}\frac{\|B_{xv_i}^{(r_{ij})}\|}{\|A_{xv_i}^{(r_{ij})}\|} - x_j x_k I_{jk}^{(i)}\frac{\|B_{xv_i}^{(r_{ij})}\|}{\|A_{xv_i}^{(r_{ij})}\|^3} \end{aligned}$$

where $I^{(i)}$ is a $(2N-2)\times(2N-2)$ dimensional matrix with $I_{jk}^{(i)} = 1$ whenever e_k is contained in $A_{xv_i}^{(r_{ij})}$ and zero otherwise. Equivalently $I^{(i)}$ indicates whether splits e_j and e_k are simultaneously contracted to zero on $\Gamma(x, v_i)$. We will show that the matrices $Q^{(i)}$ are positive semi-definite. For any vector $\xi \in \mathbb{R}^{2N-2}$ we have

$$\sum_{j,k} Q_{jk}^{(i)}\xi_j\xi_k = \sum_j \frac{b_j}{a_j}\xi_j^2 - \sum_{j,k}\xi_j\xi_k x_j x_k \frac{b_j}{a_j^3} I_{jk}^{(i)} \tag{S1}$$

where $a_j = \|A_{xv_i}^{(r_{ij})}\|$ and $b_j = \|B_{xv_i}^{(r_{ij})}\|$. Now fix a single set of splits $A_{xv_i}^{(l)}$ and let J_l denote the indices of splits in this set. If we restrict the right-hand side

of the last equation to indices $j \in J_l$ we obtain

$$\sum_{j \in J_l} \frac{b_j}{a_j} \xi_j^2 - \sum_{j,k \in J_l} \xi_j \xi_k x_j x_k \frac{b_j}{a_j^3}.$$

The terms a_j adopt the same value for all $j \in J_l$, and similarly for b_j, so they are independent of the summation index in the last expression. Also for $j \in J_l$

$$a_j^2 = \|A_{xv_i}^{(l)}\|_x = \sum_{m \in J_l} x_m^2.$$

Then

$$\sum_{j,k \in J_l} Q_{j,k}^{(i)} \xi_j \xi_k = \sum_{j \in J_l} \frac{b_j}{a_j} \xi_j^2 - \frac{\sum_{j,k \in J_l} \xi_j \xi_k x_j x_k}{\sum_{m \in J_l} x_m^2} \frac{b_j}{a_j}.$$

The Cauchy-Schwartz inequality shows the right-hand side is ≥ 0 since a_j, b_j are constant over this range of j. It follows that the right-hand side of equation (S1) is ≥ 0, so each matrix $Q^{(i)}$ is positive semi-definite. If we drop the assumption that $\mathcal{C}_{ij} = 0$ for all i, j, this introduces rows and columns of zeros into each matrix $Q^{(i)}$. However, the matrices $Q^{(i)}$ must therefore remain positive semi-definite, and this establishes the claim in the statement of the proof. □

It is relatively straightforward to prove the weaker result that the matrices with elements $\partial F_j / \partial x_k$ are positive semi-definite. The functions $f_i : \mathcal{T}_N \to \mathcal{R}$ defined by $f_i(x) = d(x, v_i)$ are convex (Bridson & Haefliger, 2011, Proposition 2.2). It follows that the objective function Ω is convex, since it is a convex combination of convex functions. Then, within the interior of any orthant, the matrix of second derivatives of Ω with respect to the edge lengths must be positive semi-definite. However, invertibility of the matrices is required in order to establish Theorem 1 in the paper, and this requires the stronger result stated in Lemma 2.

References

Bridson, M. R. & Haefliger, A. (2011). *Metric Spaces of Non-Positive Curvature.* Springer, Berlin.
